# Clavulanic Acid Production by *Streptomyces clavuligerus*: Insights from Systems Biology, Strain Engineering, and Downstream Processing

**DOI:** 10.3390/antibiotics10010084

**Published:** 2021-01-18

**Authors:** Víctor A. López-Agudelo, David Gómez-Ríos, Howard Ramirez-Malule

**Affiliations:** 1 Escuela de Ingeniería Química, Universidad del Valle, A.A., Cali 25360, Colombia; viclopezag@gmail.com; 2 Grupo de Investigación en Simulación, Diseño, Control y Optimización de Procesos (SIDCOP), Departamento de Ingeniería Química, Universidad de Antioquia UdeA, Calle 70 No. 52-21, Medellín 050010, Colombia; dandres.gomez@udea.edu.co

**Keywords:** clavulanic acid, *Streptomyces clavuligerus*, systems biology, strain engineering, downstream processing

## Abstract

Clavulanic acid (CA) is an irreversible β-lactamase enzyme inhibitor with a weak antibacterial activity produced by *Streptomyces clavuligerus* (*S. clavuligerus*). CA is typically co-formulated with broad-spectrum β‑lactam antibiotics such as amoxicillin, conferring them high potential to treat diseases caused by bacteria that possess β‑lactam resistance. The clinical importance of CA and the complexity of the production process motivate improvements from an interdisciplinary standpoint by integrating metabolic engineering strategies and knowledge on metabolic and regulatory events through systems biology and multi-omics approaches. In the large-scale bioprocessing, optimization of culture conditions, bioreactor design, agitation regime, as well as advances in CA separation and purification are required to improve the cost structure associated to CA production. This review presents the recent insights in CA production by *S. clavuligerus*, emphasizing on systems biology approaches, strain engineering, and downstream processing.

## 1. Introduction

The accessibility to effective treatment alternatives of infectious diseases depends on the availability of appropriate antibiotic compounds in the market. Efficient antibiotic production is crucial for health systems worldwide, especially in outbreaks, epidemics, and health emergencies, in which the antibiotic supply chain can be put under pressure.

Additionally, the acquired antibiotic resistance phenomenon became a global concern as it may increase the vulnerability of health systems [1]. The antibiotic resistance phenomenon emerged along with the antibiotic era [2,3]. Years before penicillin was used at global scale, a penicillinase enzyme able to inactivate penicillin was discovered in bacteria extracts [4]. Antibiotic resistance has forced humanity to maintain an endless search for new and more powerful antibiotics. In this regard, the pharmaceutical industry plays a key role in the development of effective treatments against such multidrug-resistant bacteria [1]. Since the discovery of benzylpenicillin in the 1920s, the class of compounds referred to as β-lactam antibiotics has been the most extensively used antibiotics. Nevertheless, a significant number of different antibiotic compounds (such as carbapenems, cephamycins, cephalosporins, and monobactams) has been developed and implemented in the clinical practice as a strategy to evade the acquired resistance [5]. The new combinations of antibiotics are aimed to increase their spectrum of activity and overcome the resistance barriers developed by the bacteria. In order to mitigate the bacterial resistance to β-lactam antibiotics, several compounds have been identified as β-lactamase inhibitors. Those compounds can irreversibly inactivate the β-lactamases allowing the β-lactam antibiotics to act against the infection. The main β-lactamase inhibitors are Sulbactam, Tazobactam, and clavulanic acid (CA) as clavulanate salt. 

**Figure 1:**
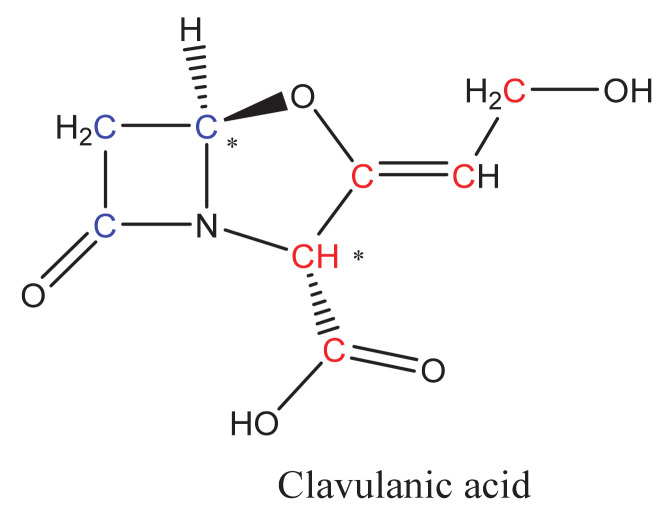
Clavulanic acid (CA) structure. Red and blue C atoms correspond to those coming from C-3 and C-5 precursors, respectively. * Stereochemical centers on CA structure.

CA is a β-lactam compound with modest antibiotic activity but high inhibition capacity of β-lactamase enzymes. The CA molecule is an analog of the penicillin nucleus, in which the characteristic sulfur atom has been substituted by an oxygen atom. CA is one of the so-called “clavam metabolites” produced by the filamentous bacterium *Streptomyces clavuligerus* (*S. clavuligerus*); most of those metabolites have the characteristic fused bicyclic β-lactam/oxazolidine ring. Nevertheless, the CA molecule (Figure 1) has 3R, 5R stereochemistry, opposite to the 3S, 5S configuration present in other clavam metabolites, which do not exhibit β-lactamase inhibition activity, although some of them have antibacterial or antifungal properties [6]. In addition to the stereochemistry, the inhibitory effect of CA has been explained by the presence of the β-lactam/oxazolidine ring that bonds irreversibly with a serine residue in the catalytic center of the β-lactamase enzyme, thus rendering it inactive [7]. Currently, CA is used in combination with other β-lactam antibiotics as an effective treatment against several clinical syndromes including pneumonia and exacerbations of chronic obstructive pulmonary disease, complicated intra-abdominal infections, acute infectious diarrhea, urinary tract infections, pharyngitis, surgical, wound, and skin infections [8]. Some of them are caused by resistant pathogenic bacteria already included in the World Health Organization priority list: *Escherichia coli*, *Staphylococcus aureus*, *Neisseria gonorrhoeae*, *Streptococcus pneumonia*, and all *Enterobacteriaceae* and *Klebsiella* species [1].

**Figure 2:**
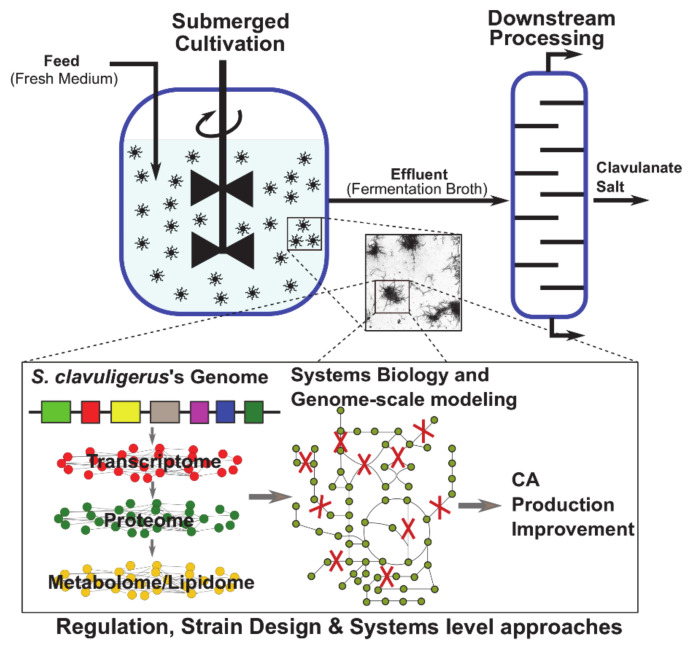
Holistic overview of the CA production process.

CA is produced worldwide at large scale by several pharmaceutical companies, and it is also prescribed in more than 150 countries [9]. CA has a relatively limited market availability and a middle–high cost for the health system, especially in the developing countries when compared with the income level, being quite inaccessible for people without health insurance. The cost of CA is mainly related to the complexity of the production process, the current uncertainties about the regulatory elements controlling the CA biosynthetic gene cluster and the intellectual property associated with its production [6]. Despite the significant number of studies related to CA production in *S. clavuligerus* submerged cultivations, low titers (~1 g∙L^−1^) are still obtained when using a wild type strain. The productivity of CA production bioprocess is also compromised by the downstream processing: CA separation from fermentation broths and precipitation as clavulanate salt. This review presents a holistic overview of CA production process: CA biosynthesis; CA production in *S. clavuligerus* submerged cultivation; recent advances in strain engineering; and elucidation of regulatory elements controlling CA production, systems biology approaches, and downstream processing (Figure 2). 

## 2. Overview of CA Biosynthesis in *S. clavuligerus*

The *Streptomyces* genus produces a wide variety of secondary metabolites with antimicrobial activity (approximately two-thirds of which occur naturally) [10]. In 1971, Nagarajan et al. [11] reported a new Streptomycete species as producer of two cephalosporin compounds. This new species was then named and described as *S. clavuligerus* by Higgens and Kastner, also in 1971 [12]. Later, in 1976, Howarth and Brown [13] described the CA chemical structure, which was elucidated via spectroscopic and X-ray analyses and reported as a novel fused β-lactam compound with a significant inhibitory activity of β-lactamases. In 1977, Reading and Cole described the cultivation conditions of *S. clavuligerus* to produce CA and the spectrometric method for CA detection [7].

In 1941, the biochemist Selman Waskman described the most accepted definition of “antibiotic” as a small molecule made by microorganisms that inhibits the growth of another microorganism. During the “golden age” of antibiotics, approximately 70–80% of the antibiotics discovered came from Streptomycetes, but the evolutionary reason for the development of antibiotic biosynthetic capacity of soil bacteria and its ecological role are still unknown. Soil bacteria are not very efficient at up-taking nutrients, and their growth rate is considerably low in comparison with other bacteria and fungi. A plausible hypothesis that iswidely accepted implies that antibiotics secretion allows the producer to control the organisms competing for the same nutritional resources in a hostile multispecies environment [2,14]. This is consistent with the secondary nature of antibiotics secretion under nutritional restriction. However, the antibiotic compounds at very low concentrations can modulate the transcriptional profiles of target bacteria and the products of resistance genes would silence those messages, opposing the theory of the development of attack and defense mechanisms [14]. Mathematical estimations point out that Streptomycetes are able to produce around 100,000 antibiotics and only 3% of them have been discovered [15]. Similarly, the existence of cryptic biosynthetic gene clusters (BGCs) suggests a higher capacity of antibiotic production in those organisms [16,17,18]. 

CA is secreted as a secondary metabolite, also referred as a specialized metabolite, by *S. clavuligerus* under nutritional restriction. The role of CA in the physiology and adaptation of *S. clavuligerus* is also unknown. As a β-lactamase inhibitor, CA may be part of a self-resistance mechanism involving the synthesis of β-lactam antibiotics, β-lactamase enzymes, and β-lactamase inhibitors; possibly developed by *Streptomyces* species to defend themselves from the effect of the antimicrobials [10]. CA biosynthesis is induced during phosphate limited conditions; recently it has been proposed that CA production may be a consequence of a homeostatic response aimed to compensate for the ATP deficit under phosphate depletion [19]. This mechanism would trigger a strong activation of oxidative and amino acid metabolism, producing reduced cofactors and ATP and favoring the antibiotic biosynthesis as a feasible mechanism to adjust the ATP generation [20,21]. Moreover, the simultaneous secretion of penicillin and cephalosporins antibiotics along with β-lactamase enzymes and β-lactamase inhibitors would tightly control the energetic impact of an ATP-consuming futile cycle of polymerization/degradation of the cell wall caused by the β-lactam antibiotics accumulation [19,22,23].

**Figure 3:**
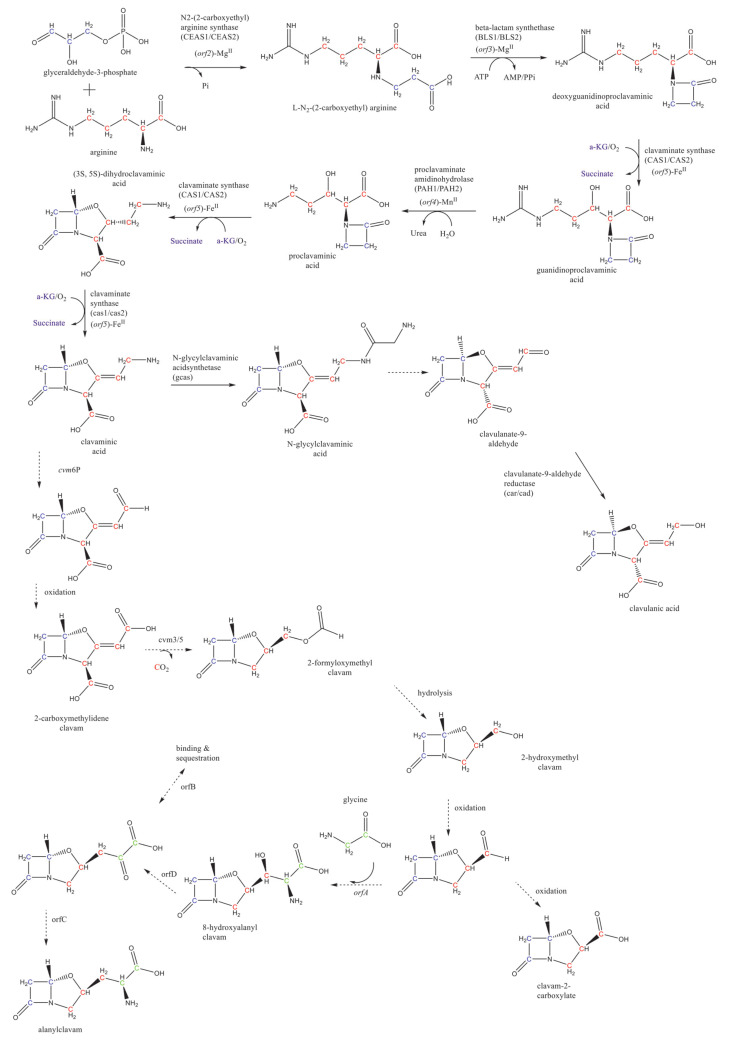
Summary of the CA biosynthetic pathway in *S. clavuligerus*. The green, blue, and red Cs represent the carbon atoms coming from metabolic intermediates such as glycine, glyceraldehyde 3-phosphate, and L-arginine, respectively. Adapted from the work in [43].

CA is a product of the clavam pathway, which proceeds towards two branches: the CA and the clavams 5S biosynthesis [6]. The clavam pathway contains two sets of reactions: the so-called “early” and “late” steps. The early steps start with the condensation of the glycolysis intermediate glyceraldehyde-3-phosphate and the amino acid L-arginine, producing the *N*^2^-(2-carboxyethyl)-arginine. This condensation is catalyzed by the *N*^2^-(2-carboxyethyl)-arginine synthase (CEAS). The early steps comprise five well-known reactions leading to the (3S, 5S)-clavaminic acid. The formation of the clavaminic acid is considered a bifurcation point of the carbon flowing through the pathway. The CA biosynthesis late steps consider the reactions leading to CA, as well as the reactions forming the clavam 5S compounds after the clavaminic acid bifurcation [24,25,26]. The known reactions of the clavam pathway are summarized in Figure 3. The β-lactam compound deoxyguanidinoproclavaminic acid is synthesized from the *N*^2^-(2-carboxyethyl)-arginine in the second reaction of the clavam pathway, catalyzed by the β-lactam synthetase (BLS) [27,28,29]. The hydroxylation of the deoxyguanidinoproclavaminic acid takes place to form the guanidinoproclavaminic acid; a reaction mediated by clavaminate synthase (CAS) [29,30,31,32]. Subsequently, the proclavaminic acid is produced by removing the amidino group from the arginine residue on the guanidinoproclavaminic acid, by action of proclavaminate amidino hydrolase (PAH) [33,34]. The dihydroclavaminic acid is obtained from the proclavaminic acid through oxidative cyclization and desaturation, both catalyzed by CAS, leading to the (3S, 5S)-clavaminic acid [29,35]. At this point, the late steps start with the carbon flux bifurcation in two branches: one branch leading to the clavulanate-9-aldehyde and further the CA, and the other branch forming clavam 5S compounds. It is important to highlight that the 3S, 5S stereochemistry of clavaminic acid is conserved in the synthesis of all clavam 5S compounds. In the case of the CA, a transition from 3S, 5S configuration to 3R, 5R is required. Some authors have suggested that this stereochemical inversion may occur via the N-glycyl-clavaminic acid intermediate to form the clavulanate-9-aldehyde with a 3R, 5R configuration, and therefore the CA [36,37]. However, more experimental evidence is required for the complete elucidation of intermediate reactions connecting the clavaminic acid with the clavulanate-9-aldehyde [36,37], which is lastly reduced to CA by the action of clavulanate dehydrogenase (CAD) [38]. Recently, Gómez et al. [39] proposed six reaction steps for the 3S, 5S to the 3R, 5R transition, by using a computational approach. 

In addition to the CA, other metabolites have been identified as side compounds of this biosynthetic pathway, namely, N-glycyl-clavaminic acid, N-acetylglycyl-clavaminic acid, and N-acetyl-clavaminic acid. It has been suggested that those compounds result from the intermediate steps involved in the transition of the 3S, 5S stereochemistry into the 3R, 5R of CA [36]. Additionally, several metabolites (2-hydroxymethylclavam, 2-formyloxymethylclavam, clavam-2-carboxylic acid, and alanylclavam) have been identified and grouped as clavam 5S compounds due to their 3S, 5S stereochemistry [37,40,41]. Despite their structural similarity and common precursors, only the clavam compounds with a bicyclic nucleus formed by the β-lactam ring and an oxazolidine ring with 3R, 5R stereochemistry can effectively inhibit the β-lactamases [10,40]. 

Glycerol is the main substrate in the CA production by *S. clavuligerus* as it has direct incorporation into the glycolytic pathway by forming glyceraldehyde 3-phosphate, the first C-3 precursor of CA [42,43]. Then, the carbon flux splits into three pathways, two of them belonging to the primary metabolism: glycolysis and gluconeogenesis, and one belonging to the secondary metabolism: the clavam pathway [43,44,45]. In addition to glyceraldehyde 3-phosphate, the need for a C-5 precursor implies the constant demand for L-arginine. This amino acid is synthesized in the urea cycle while L-glutamate and L-aspartate promote its biosynthesis by fueling the urea cycle in the oxidative direction [42,43,44]. 

## 3. CA Production in *S. clavuligerus* Submerged Cultivation

Besides CA, *S. clavuligerus* produces a plethora of secondary metabolites that can compete with CA biosynthesis and secretion. Those include some β-lactam (penicillin N, cephamicin C, cephalosporins, and other clavams) and non-β-lactam (holomycin, β-lactamase inhibitory protein and a tunicamycin-related antibiotic) antibiotics [24,46,47,48,49,50,51,52,53,54]. However, CA is currently the most important specialized metabolite produced by *S. clavuligerus* considering its clinical and industrial relevance. 

CA is usually produced in submerged cultivation of *S. clavuligerus*, either wild type or engineered strains. Several operation modes and media compositions have been explored aimed to increase the characteristic low titters of the wild type strain, as Ser et al. [55] reviewed recently. Such strategies might also enhance the CA titers of engineered strains in submerged cultivation. As previously mentioned, glycerol is the most common carbon source used in CA production. When comparing the CA production using glycerol and starch as carbon sources, glycerol promotes CA titers up to 5-fold the observed in cultivations with starch as carbon source [56,57]. Nevertheless, substrate inhibition can occur at glycerol concentrations higher than 50 g∙L^−1^ [56]. As glycerol acts as the C-3 precursor, the biosynthesis of CA is enhanced by amino acid supplementation [42,58] and rich nitrogen sources like soy protein and soy flour [56,57] that provide abundant C-5 precursors through the urea cycle. Conversely, the use of dextrose or starch promote the secretion of Cephamycin C [57,59]. In a media screening study, Da Silva et al. [60] observed the highest CA production (0.437 g∙L^−1^) with the media having glycerol as the main carbon source and isolated soy protein, while the amino acid supplementation did not enhance the CA productivity. Neto et al. [61] reported CA concentrations in batch, fed batch and continuous cultivations of 0.195, 0.404, and 0.293 g∙L^−1^, respectively. The highest CA concentrations obtained in bioreactor cultivations using a wild type strain were reported by Teodoro et al. [62]. In that study, batch and fed-batch operation modes were tested using a complex medium prepared with glycerol, malt, and yeast extracts, peptone and trace elements. Batch cultivations yielded 0.430–0.530 g∙L^−1^ of CA while fed-batch cultivations with ornithine (3.7 g∙L^−1^) led to a CA concentration of 1.560 g∙L^−1^ [62,63]. A complex culture medium provides important amounts of free or hydrolyzed amino acids, which act not only as nitrogen sources, but also as secondary carbon sources. Complex nitrogen sources, such as those containing soy protein, favor considerably the CA accumulation in contrast to the chemically defined media [56]. The use of complex media with vegetable oils supplementation (olive, cotton, palm, and corn) as secondary carbon sources may enhance the CA production up to 2-fold when compared to glycerol as a supplement [64,65,66]. However, the use of complex media leads to denser and more viscous fermentation broths due to the high concentration of suspended solids. Those factors may affect the mass transfer in the bioreactor and the downstream CA separation. Phosphate has a potential repressive effect on CA production; thus, culture media are commonly designed as phosphate-limited [6,42,55]. In this regard, Saudagar and Singhal [58] found a CA biosynthesis repression when increasing the phosphate concentration in the media over 100 mmol.L^−1^. The studies indicated that CA accumulation is favored in the fed-batch operation under controlled conditions of pH, aeration, and stirring. Those conditions are more easily controllable in stirred tank bioreactors (STR) although the CA production studies are not restricted only to this configuration.

Other factors apart from culture media could affect the CA accumulation in submerged cultures of *S. clavuligerus*. The CA molecule shows high susceptibility to temperature and ionic strength in aqueous solutions, compromising the CA titers during the cultivation [67,68]. The pH conditions seem to affect CA yield, possibly more linked to molecule degradation than biosynthesis inhibition [69]. The highest concentration of CA has been reported at neutral or slightly acidic pH conditions (6.8), regardless of the substrates used in the cultivations [55]. A recent report showed that STR cultures of *S. clavuligerus* subjected to acid stresses (pH reduction from 6.8 to 6.3 at a rate of 0.1 pH every 6 h) may lead to higher global yields ($Y_{\mathrm{CA}/X}=0.851{\;g}_{\mathrm{CA}}.g_{x}{}^{-1})$ [70]. Low temperatures favor the molecule stability; in this regard, higher titers have been reported during operating fermentations at 20 °C but the growth rate at such temperature is lower than that observed at 28 °C [70,71]. In addition, it has been found that both bacterial morphology in dispersed secondary mycelium and a high density spore suspension inoculum (with concentration ≥5%) significantly improve CA biosynthesis [72,73,74].

Regarding the operational variables associated to fermentative processes in *Streptomyces* species, the nutritional effects have been more studied than the effects of hydrodynamic conditions and reactor geometry [75]. The conventional bioprocesses with *Streptomyces* strains are generally carried out in STRs, as this traditional geometry has been proven to be reliable as it assures good mixing, mass, and heat transfer rates [76]. Novel impeller geometries for STR have been studied as feasible alternatives for improving the oxygen dissolution and nutrient dispersion during the fermentative process [77]. Few studies involving the hydrodynamic patterns of reactors and its impact on growth, morphology, and mass transfer in *Streptomyces* cultures have been performed. Such studies are required for a more precise description of antibiotics biosynthesis in bioreactors [75]. The reactor geometry, impeller configuration, and velocity of mixing may impact the oxygen availability on the system; it has been demonstrated that higher mixing velocities and turbulence gives better volumetric oxygen mass transfer coefficients (*k_La_*), promoting higher specific growth rates [76,78]. The reactor design must be focused on balancing mass and heat transfer with shear rates, as the latter can cause hydrodynamic stress on cells, affecting their growth and productivity. In this direction, the novel single-use technologies can offer some advantages, in addition to the better sterility conditions attained in this kind of reactors [75]. 

In the operation of bioreactors, the agitation rate is related not only with the dissolved oxygen and mass transfer conditions, but also with the shear conditions. The shear forces may affect the broth rheology and transport of nutrients as well as the growth rate and morphology expression on *Streptomyces* species [79,80]. In the case of *S. clavuligerus*, agitation and aeration are also considered as factors potentially affecting the CA production. An increase of 50% in oxygen transfer in shake flask cultures increased the CA production 2-fold [81]. Regarding the bioreactor operation, Rosa et al. [82] found that intense agitation rates, i.e., 800–1000 rpm, favored CA accumulation in STRs. The *k_La_* observed in airlift reactors, operated in the range from 3 to 5 vvm, are rather comparable to those arising in STRs operating in the range of 600 to 1000 rpm; both conditions promote CA accumulation. An analysis of the *S. clavuligerus* response to shear stress conditions showed that high shear forces (up to 7.5 Pa.s) increase the growth rate, mycelial thinning, and CA production, in comparison with cultivations at low shear forces (<1 Pa.s) [79]. Moreover, the mycelial diameter increased 57% under low shear forces when compared to the morphology at high shear stress (1.44 µm); suggesting that CA productivity might be affected by the surface–volume ratio, and consequently by the transport rate of nutrients, oxygen, and product release [79]. In summary, high CA concentrations are attained under adequate provision of C-3 and C-5 precursors that increase the carbon flux towards the biosynthetic pathway under phosphate limitation, while maintaining slightly acidic conditions, controlled temperature, high shear rates, and aeration. Such conditions promote both high biomass production and high CA secretion. Rich media are preferred due to their low cost, availability, and high amino acids content, but a proper C/N balance must be maintained along with the phosphate restriction; otherwise, low CA titers could be obtained.

CA chemical synthesis has been also explored as an alternative production method. In this regard, at least two different ways for the synthesis of CA have been reported by using diester and diene compounds [83,84]. Nevertheless, the mentioned approaches yield low concentrations of racemic mixtures of CA methyl esters. The products and yields of CA organic synthesis imply more complex and costly separation procedures than in case of the bioprocess. Thus, CA production via submerged cultivation with *S. clavuligerus* has been selected by the pharmaceutical industry as the more profitable alternative, given the stereochemical selectivity of the biochemical pathway. Likewise, research has focused on solving the key issues in the bioprocess instead of the chemical synthesis, namely, the low productivity of the strain, the susceptibility to hydrolysis, and the efficiency of the separation/concentration processes.

## 4. Regulation of the CA Production in *S. clavuligerus*

Successful secondary metabolite biosynthesis by the *Streptomyces* genus undergoes complex regulation at many levels, involving transcriptional regulators (pleiotropic regulators and pathway-specific regulators), sigma-factors, and small non-coding RNAs [85]. Therefore, the system-level understanding of the complex regulatory interactions and signaling pathways may lead to novel improvement strategies in the CA production. 

Usually, antibiotic biosynthetic clusters are regulated by two types of transcriptional regulators: pathway-specific regulators (PSRs) and pleiotropic regulators [86]. At a lower level, PSRs are located inside the antibiotic biosynthetic cluster and these proteins can bind directly to the promoters of genes in the same biosynthetic pathway [87,88]. Similarly, at the top level, global regulators are known to control the production of antibiotics in the *Streptomyces* genus [87,88]. 

The *claR* is a PSR that belongs to the LysR family and triggers the late genes (*cyp, oppA1*, and *cad*) of the CA biosynthetic pathway, specifically those participating in the reactions of CA and clavaminic acid biosynthesis [85,89]. Furthermore, the *ccaR* is a PSR located in the adjacency of the cephamycin C cluster, and triggers the synthesis of both CA and cephamycin C. The last compound is induced by the *ccaR* through the regulation of the early genes *cas2*, *bls*, *pah*, and *orf2* [24,85,90]. The evidence suggests that both *ccaR* and *claR* form a regulatory system that controls the CA biosynthesis [91]. Recently, by means of a comparative transcriptomic analysis, Pinilla and colleagues [92] identified that CA cluster genes were up regulated, including both *ccaR* and *claR*, when *S. clavuligerus* was cultured in complex media.

As the CA titers in cultivations using the wild type strain are still low, engineered strains of *S. clavuligerus* perturbing these regulators have posed as attractive alternatives for improving the CA production. Pérez-Llarena and co-workers [93] found that a deletion mutant of *ccaR* is unable to synthesize CA and cephamycin C; besides, an amplification of this gene led to 2–3-fold rise in the CA and cephamycin C biosynthesis. Furthermore, Alexander and Jensen [94] investigated the role of the CcaR protein in the cephamycin C gene cluster in *S. clavuligerus*. The *ccaR* gene was also considered as essential in the synthesis of CA, cephamycin C, and non-CA clavams [94]. The authors also concluded that the lack of cephamycin C production in *ccaR* mutants was mainly due to the absence of some enzymes in the early and middle steps in the cephamycin C pathway. Pérez-Redondo et al. [89] observed that high replication of the *claR* gene in multicopy plasmids led to 3-fold and 5–6-fold increases in the CA and alanyclavam production, respectively, with a consequent reduction of cephamycin C production.

Furthermore, Kizildoğan and co-workers [95] engineered a CA-overproducer (*S. clavuligerus* IDG3) strain yielding CA titers up to 6.690 g∙L^−1^. In that study, the authors overexpressed clavaminic acid synthase (*cas2* gene) and the genes *claR* and *ccaR* independently using diverse promoters that were introduced into the industrial strain *S. clavuligerus* DEPA. The overexpression of *ccaR* under the control of a *glpF* promoter, introduced into *S. clavuligerus* DEPA, led to 25.9-fold CA production compared to its vector control. Qin et al. [96] fused the *neo* gene (a kanamycin resistance gene without promoters) with *claR*, generating the *S. clavuligerus* (NEO) strain able to produce CA titers up to 3.260 g∙L^−1^. Subsequently, and after a series of treatments and screening, the authors obtained a new engineered strain (M3-19), which produced up to 4.330 g∙L^−1^ of CA, an increase of 33.8% compared to the NEO strain. Recently, Cho et al. [97] overexpressed the genes *ccaR, cas1*, and *claR* in the mutant strain *S. clavuligerus* OR. Upregulation of *cas1* improved the CA yield in *S. clavuligerus* OR, leading to CA concentration of 4.95 g∙L^−1^, approximately 25% more compared to the control *S. clavuligerus* OR. Additionally, the overexpression of *claR and ccaR* in the same mutant strain increased the CA accumulation up to 5.66 g∙L^−1^ in shake flask cultivations. Nevertheless, a co-expression of *claR, ccaR*, and *cas1* did not improve the CA titers. Batch cultivation of *S. clavuligerus* OR with overexpressed ccaR and claR in a 7 L bioreactor accumulated 6.010 g∙L^−1^ of CA.

In addition to *claR and ccaR*, the regulatory pathway that controls CA production contains other important regulators. The CcaR formation is modulated by other regulatory proteins like AreB and ScaR, both γ-butyrolactone receptor proteins. AreB is an autoregulation binding protein that belongs to the transcriptional regulators family termed IclR and is located upstream of the *ccaR* gene; it binds to the sequences (ARE, autoregulatory elements) located upstream of target genes [24]. Santamarta and colleagues [98] found that AreB modulates the assimilation and biosynthesis of lysine. The mutant *∆areB* of *S. clavuligerus* induces an increase in CA and Cephamycin C levels; highlighting an important role of *areB* in connecting secondary and primary metabolism. ScaR (Brp, γ-butyrolactone receptor protein) is an autoregulator induced by γ-butyrolactone that controls directly *ccaR* and indirectly *claR* via the AdpA signaling pathway. The AdpA transcriptional regulator is downregulated by ScaR (in *scar* mutants *adpA* is upregulated), and regulates positively the CA production, as it was demonstrated by the mutant *∆adpA* strain [99].

In the *Streptomyces* genus, an important type of global regulators is the two-component system (TCS), which usually regulates both antibiotic production and morphological development. The TCS consists of a histidine kinase bound to the extracellular membrane, which senses external perturbations, and a response regulator, which triggers the transcription of target genes by undergoing a signal transduction pathway [100,101]. Fu and colleagues [102,103] found that CagRS is a pleiotropic regulatory TCS that has a positive effect on CA production. Transcriptome analysis of the mutant *∆cagRS* versus wild type shown that CagRS TCS induces the up regulation of the CA biosynthetic gene pathway and also that CagRS positively regulates glyceraldehyde 3-phosphate but negatively regulates arginine biosynthesis [102]. 

Similarly, other TCS have been identified for *S. clavuligerus*. Kwong and colleagues [104] found that the transcription of the transcriptional activator *cvm7p* is controlled by a TCS formed by *snk* and *res2*. Mutants of *res2* and *snk* were incapable to synthesize metabolites of the 5S clavams pathway [104]. Another important two-component regulatory system is associated with the phosphate (Pho) regulon, the unique known regulatory mechanism in response to phosphate starvation in bacteria [105]. The hypothesis of a Pho regulon present in *S. clavuligerus* is supported by the high production of CA under phosphate limitations. Salehghamari et al. [106] predicted the existence of this regulon by the identification of PHO binding sequences in promoter regions of 31 genes of *S. clavuligerus*. 

Likewise, other TCS is the AfsK-AfsR serine/threonine kinase system, which induces the production of secondary metabolism gene clusters in *S. lividans* and *S. coelicolor* [107]. The *asfR-p* is a homologue of *asfR*, the substrate of AfsK from the AfsK-AfsR. Paraluji and colleagues [108] demonstrated the potential of this pleiotropic regulation by overexpressing *asfR-p* in distinct *Streptomyces* bacteria, including *S. clavuligerus*, which showed a CA production of 0.065 g∙L^−1^. 

Recently, it was found that CepRS (cephamicyn regulator/sensor) is a TCS regulator with a key role in the cephamycin C biosynthesis [109]. Although this TCS did not affect the production of CA, this mutant strain (*∆cepRS*) in combination with the overexpression of CA specific regulators may be an interesting alternative for improving CA production.

Expression of secondary metabolism in bacteria is also regulated by sigma factors. Sigma factors are proteins that recognize the RNA polymerase and allow the proper union to the gene promoter, leading to the synthesis of a mRNA molecule [110]. Similarly, an anti-sigma factor inhibits the transcription, as they will bind to the RNA polymerase, thus preventing that sigma factors from mediating the binding to the promoter site [110].

The sigma factor gene, *orf21*, is located downstream of the CA biosynthesis cluster in *S. clavuligerus*. Jnawali et al. [111] disrupted and overexpressed this gene in a *S. clavuligerus* NRRL3585 and found a reduction 10–15% in the levels of CA in the *∆orf21* mutant; while in the same conditions, overexpression of *orf21* exhibited a 1.45-fold increase in CA yields during fermentation. *S. clavuligerus* also encodes *bldG*, an anti-anti-sigma factor, which controls genes (regulated by sigma factors) involved in morphological development and secondary metabolite biosynthesis. Bignell and colleagues [90] found that *bldG* regulates CA biosynthesis by modulating the expression of *ccaR*, and recently, this regulator has been associated with a direct regulatory role in the tunicamycin biosynthesis [112].

The stringent response is a stress response phenomenon that occurs in bacteria under substrate starvation, e.g., amino acid deprivation, iron restriction, and other stringent conditions [113]. This stress mechanism involves the RelA/SpoT protein system (ppGpp synthase (RelA) and (p)ppGpp synthase/hydrolase (SpoT)), which mediates the synthesis of phosphorylated nucleotides like (p)ppGpp [24,85]. This nucleotide exerts a regulatory role in ribosomal genes via RNA Polymerase binding it is also involved in the morphological differentiation of *S. clavuligerus* [114]. Likewise, a *relA* mutant of *S. clavuligerus* showed reduced capability to yield both CA and cephamycin C [115].

In addition to the strain design based on overexpression or mutation of regulatory genes, some research efforts have been focused in targeting the primary metabolism. Li and Townsend [116] doubled CA production by deleting the *gap1* gene in a mutant of *S. clavuligerus* NRRL3585, increasing the glyceraldehyde 3-phosphate pool for CA biosynthesis. Jnawali et al. [117] used the last approach along with the overexpression of ccaR and claR to create an overproducer engineered strain. Recently, overexpression of *lysA* in *S. clavuligerus* NRRL 3585, a gene involved in L-lysine biosynthesis (coding for a diaminopimelic acid decarboxylase), showed improvements in the production of CA, cephamycin C and tunicamycin.

In this decade, it has been also described engineered strains of *S. clavuligerus* perturbing random genes through mutagenesis techniques. Medema and colleagues [118] engineered an industrial strain (DS48802) through an iterative process of mutagenesis and screening of *S. clavuligerus* ATCC 27064. This mutant strain produced CA levels 100 times more than the wild type. Likewise, an improvement in CA yields using mutagenesis induced by ultraviolet on *Streptomyces sp. NRC77* was reported [119]. The authors found a mutant strain after screening *(Streptomyces sp. MU-NRC77*) that was able to produce 0.272 g∙L^−1^ of CA in the production medium, while the addition of H_2_O_2_ and plant charcoal increased the production up to 0.649 and 0.683 g∙L^−1^, respectively. Recently, Cruz-Hernandez et al. [120] also used UV mutagenesis for enhanced CA titer by *S. clavuligerus*. The authors obtained mutants of *S. clavuligerus* ATCC 27064 via UV radiation, and one of them achieved a CA concentration of 0.500 g∙L^−1^. Although both studies reported an increase of 5.2- and 1.6-fold on CA production compared to wild-type strain, respectively, the titers are too low to be considered in a large-scale process. The summary of studies reporting the highest CA titers using *S. clavuligerus* engineered strains is presented in Table 1.

Despite *S. clavuligerus* being the strain commonly used in the large-scale process of CA production, there are other CA-producer *Streptomyces* species, which might be further studied and possibly engineered.

## 5. Advances in Systems Level Understanding of the CA Production in *S. clavuligerus*

In the recent decade, whole system-level approaches and omics technologies are being developed to explore and understand the existing complex interaction between different biological layers, such as the genome, transcriptome, proteome, and metabolism. A deeper understanding of the BGCs regulation and expression in *S. clavuligerus* is important to improve the CA production in submerged cultures. 

The first draft genome of *S. clavuligerus* was sequenced by two independent research groups in 2010 [121,122]. The genome is composed of a 6.8 Mb chromosome and four linear plasmids named pSCL1 (11 kb), pSCL2 (150 kb), pSCL3 (430 kb), and pSCL4 (1.8 Mb), and a GC% of 72. The global annotation of this genome refers six rRNA operons, 66 tRNA genes, and 7898 protein-coding genes (5700 in the chromosome). The *S. clavuligerus* genome contains a plethora of BGCs that includes secondary metabolites like staurosporine, moenomycin, terpenes, pentalenenes, phytoenes, siderophores, lantibiotics, holomycin, cephamycin C, and CA [121]. Specifically, the gene cluster of CA is situated in the chromosome and there exists a paralog gene cluster located in the pSCL4 plasmid codifying for CA and clavam metabolite production. In 2016, the genome of a *S. clavuligerus* industrial strain (F613-1) was sequenced. The assembly of the genome sequences identified a chromosome and a plasmid. The main differences were reported in the plasmid of F613-1, which contains a smaller number of coding genes compared to the 1.8 Mb megaplasmid of *S. clavuligerus* ATCC 27064 [123]. The significance of these differences needs further investigations.

Recently, Hwang and colleagues [124] performed a complete systems biology analysis for identifying new regulatory elements and BGCs in *S. clavuligerus* using new generation high-throughput technologies. In this research, the authors sequenced a high-quality genome of *S. clavuligerus* ATCC 27064, and it was used as a template to integrate the ribosome profiling and differential RNA-seq data. In that study, 58 potential BGCs were identified, including CA, 5S clavams, holomycin, tunicamycin, and 14 terpene type and 12 polyketides type BGCs. Furthermore, 2659 Transcription Start Sites (TSSs) and regulatory elements in the whole genome were identified. The same study reported that COG functional categories like coenzymes, secondary metabolites, inorganic ions, carbohydrate, and amino acid metabolism exhibited high differential expression levels during distinct growth phases while categories like signal transduction, replication, and cell division exhibited constitutive expression during all growth phases. Similarly, Lee and colleagues presented a high-quality genome and developed a data mining analysis for identifying secondary metabolite BGCs in thirty *Streptomyces* strains, including improvements in previous genome annotations and the identification of 43 BGCs in *S. clavuligerus* [125]. Similar approaches were performed for other bacteria of the *Streptomyces* genus [126].

Although the analysis and study of the transcriptome in *S. clavuligerus* have been relevant for identifying regulatory elements in the genome, they are also useful for understanding the up- or downregulation of metabolic pathways involved in CA production at specific nutritional conditions. In 2011, Medema et al. [118] developed the first comparative transcriptome analysis of a *S. clavuligerus* industrial strain (engineered by random mutagenesis) versus a wild type strain. They captured transcript changes in central carbon and secondary metabolism, specifically, upregulation of ammonium and phosphate transporters, and glutamine and glutamate synthase genes. In 2014, Álvarez-Álvarez and co-workers [127] developed a comparative transcriptomic analysis between a *ccaR*-deleted mutant and the wild type strain *S. clavuligerus* ATCC 27064. The authors observed that cephamycin C and CA biosynthetic genes were negatively regulated in the *ccaR* mutant, a gene expression pattern previously identified [128]. Likewise, *blip* and *blp*, two genes encoding β-lactamase inhibitory proteins, were downregulated. The most interesting metabolic phenotype was the overexpression of all the genes involved in holomycin biosynthesis, in agreement with previous studies reporting high holomycin production in *ccaR* mutants [129]. Similar results were found in a *claR* mutant; genes like *hlmI* and *hlmH* were upregulated during the entire fermentation [130]. In contrast, poor regulation of genes in the initial and late steps of CA biosynthesis, and thus no CA production were detected [130]. Recently, the *oppA2* gene (oligopeptide binding protein permease) was identified as essential for CA production [131]. Transcriptional analysis of *oppA2* mutant compared to the wild type strain also allowed identifying upregulation of holomycin genes and no significant expression of cephamycin C and CA biosynthesis clusters. However, the most interesting result was that *oppA2* mutant secreted N-acetylglycyl-clavaminic acid, an intermediate of the CA biosynthesis. The evidence suggests that this peptide probably is bound to the *OppA2* protein forming a multi-enzymatic complex in the final stages of CA biosynthesis [131]. A proteomic study of the same *oppA2* mutant identified high level abundance of rhodanese-like enzyme (*rhlA*, thiosulfate sulfur transferase) [132]. Deletion of this gene showed impairment of holomycin (90%) and lower CA and cephamycin C production.

Overexpression of non-native transcriptional regulators into *S. clavuligerus* could become an interesting approach for improving antibiotic production in the *Streptomyces* genus. In 2019, Martínez-Burgos et al. [133] overexpressed *pimM* in *S. clavuligerus* ATCC 27064, a PAS-LuxR activator of the pimaricin biosynthetic cluster present in *Streptomyces nataliensis*. A comparative transcriptome analysis indicated that overexpression of *pimM* triggers the production of both CA and cephamycin C, by affecting the expression of secondary metabolite gene clusters like SMC10, SMC13, and SMCp5, including the upregulation of CA biosynthesis and cephamycin C clusters [133]. Untargeted metabolic profiling of the *pimM* expression identified antifungal compounds similar to tunicamycin.

A proteomic analysis comparing the wild type strain of *S. clavuligerus* and two mutants lacking the global regulators *bldA* and *bldG* (*bldA* regulates the morphological development and *bldg* regulates CA and cephamycin C production) was conducted [134]. The authors showed that the *bldA* mutant reduced the CA production by 80%, while in the *bldG* mutant the CA levels were not detected compared to the wild type [134]. The *bldG* mutant exhibited a large percentage of differentially expressed proteins; those classified in functional categories like metabolism, cellular processes, regulation, translation, and energetics. When compared to the wild type, the *bldA* mutant showed a lower percentage of differential expressed proteins in the same categories [134]. The most interesting result of this whole-cell proteomic analysis was the detection of protein regulators (such as BldG, RshA, SigH, BldD, AdsA, BldC, AdpA, AfsA, AbsB, DraK, AfsK, and PhoP) involved in the regulatory cascade of CA biosynthesis [134]. Another whole-cell proteome analysis was developed by Ünsaldı et al. (2017). They performed a proteome-wide study in an industrial CA overproducer strain *(S. clavuligerus* DEPA) [135]. In that case, three CA biosynthetic enzymes were overexpressed: (i) *N*^2^-(2-carboxyethyl)-arginine synthase (CEAS2), (ii) clavaldehyde dehydrogenase (Car) (also known as clavulanic acid dehydrogenase (CAD)), and (iii) carboxyethyl-arginine β-lactam synthase (BLS2). The authors identified 33 different ORFs that were underexpressed and 60 ORFs that were overexpressed. A decrease on the expression level of genes associated with methionine biosynthesis and some enzymes of the glycolytic pathway were observed and identified as the main causes for the CA overproduction in the *S. clavuligerus* DEPA strain [95,135]. 

Most previous studies have, at least, one point in common: the genes manipulated are involved, or related, to the clavam pathway. However, the evidence suggests that investigations should not target only the clavam pathway to enhance CA production. Despite the recent advances regarding the cephamycin C and CA gene cluster in *S. clavuligerus*, further research on those pathways and other regulatory pathways must be conducted in order to decipher the complex metabolism of *S. clavuligerus*. In this regard, ChIP-Seq, transcriptomic, and proteomic analysis may contribute to elucidate the mechanisms involved in the “late” steps, especially the genes responsible for the inversion of (3S, 5S)-clavaminic acid into (3R, 5R)-CA, and their regulation and connection with CA biosynthesis. Additionally, more studies focused on the 5S clavam branch are required. For instance, studies related with *cvm6P* (encode a putative aminotransferase responsible for the first reaction of the 5S clavam pathway) and the carbon flux control downstream this bifurcation point, as a strategy to enhance the CA production [136].

## 6. Genome-Scale Modeling: A Suitable Platform for Improving CA Production

As previously described, the development of high-throughput techniques has allowed to measure and quantify a high number of cellular molecular constituents from the different biological levels (genome, RNAs, proteins, metabolites, etc.). The analysis of omics data is giving lights about the interrelated or individual relationship of biological levels (genes, proteins, regulators, and metabolites) and the production of specialized metabolites [137,138,139,140]. However, the integration of multiple omics datasets for analyzing the interplay between biological levels is still a challenging task [141,142,143,144,145,146]. Most of the proposed methodologies for multi-omics data integration have been focused on the so-called element-based approaches (correlation, clustering, and multivariate analysis) [147]. These are useful to define correlations or groups in a particular biological level and a specific condition, but not to reveal the molecular mechanisms associated with a specific cell phenotype. However, the use of mathematical-based approaches considering system components, network interaction, and physicochemical properties of the cell, poses as a suitable alternative for multi-omics data integration. Such approaches can be used to infer the interplay between biological levels through the identification of mechanisms governing the cell phenotype.

A mathematical-based approach that allows studying the metabolic fluxes and phenotypes of a cell under a plethora of nutritional/genetic/regulatory perturbations is the genome-scale metabolic modeling (GEMs) [148,149]. This mathematical platform along with the integration of omics data (genomics, transcriptomics, proteomics, metabolomics, etc.) has demonstrated usefulness in guiding experimental efforts and elucidating mechanisms affecting cell phenotypes in many different cell types [150,151,152,153,154,155,156]. 

Usually, GEMs contain all the biochemical knowledge of a cell through the formulation of mass balances per metabolites and the existing association between genes, proteins/enzymes, and reactions [157]. The stationary mass balance is the simplest mathematical formulation allowing computing carbon fluxes under specific nutritional conditions. The mass balances are represented in a stoichiometric matrix multiplied by an unknown vector of reaction velocities (reaction fluxes), configuring a linear programming problem with an objective function based on some evolution criteria like the maximization of growth or energy production yield [158]. Under this consideration the in silico cells use most of the available nutrients to grow as expected to occur under the appropriated biological conditions [159]. This approach is vastly known as Flux Balance Analysis (FBA) and it is widely used for studying cell metabolism in many areas of research [160,161].

**Figure 4:**
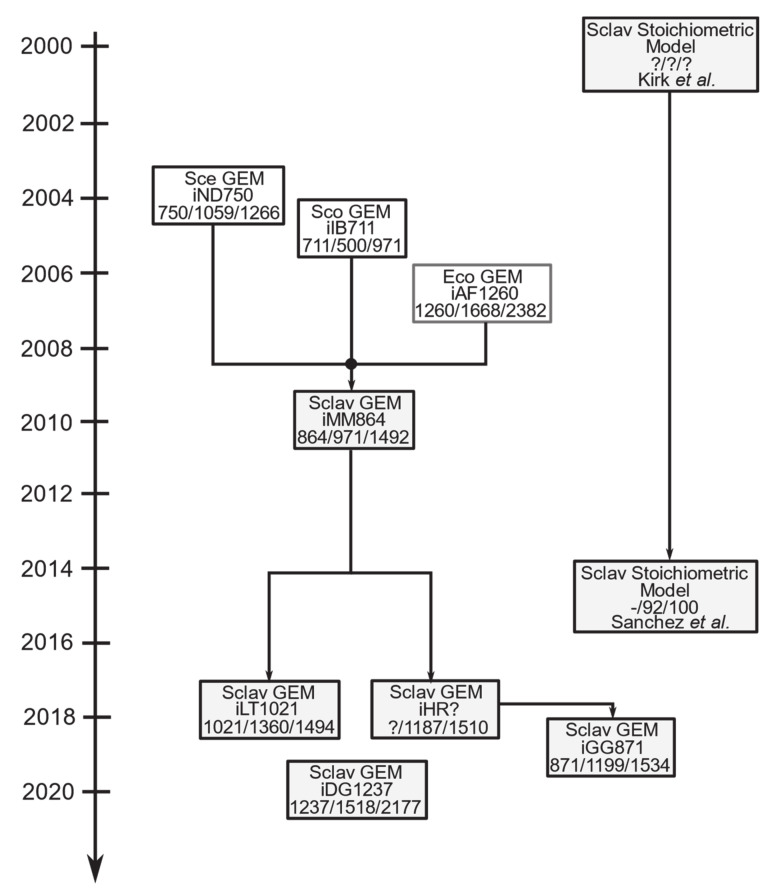
The evolution of *S. clavuligerus* metabolic models. Species abbreviations are Sclav: *Streptomyces clavuligerus*, Sco: *Streptomyces coelicolor*, Sce: *Saccharomyces cerevisiae*, Eco: *Escherichia coli*. Similarly, number of genes, metabolites, and reactions were included after a/symbol, respectively.

Application and use of genome-scale metabolic modeling of *Streptomyces* bacteria is currently an active field of study [138,145,162,163,164], including the modeling of *S. clavuligerus* metabolism (Figure 4) [19,43,122,165,166]. 

In 2000, Kirk and colleagues [167] published the first reconstructed metabolic model of *S. clavuligerus;* a mathematical model limited to central carbon metabolism, amino acid biosynthesis, anaplerotic reactions, urea cycle, and the specific pathway of CA biosynthesis. This model was used to explore the metabolic adaptation strategies of *S. clavuligerus* during a chemostat culture under phosphate, carbon and nitrogen limitations. The researchers found that anaplerotic metabolism decreases under carbon limitation, limiting the use of alpha-keto glutarate from the TCA cycle to synthesize C5 precursors. Conversely, under phosphate limited conditions the flux through C5 precursors was favored, increasing the CA production. 

In 2010, Medema and colleagues [122] used the annotation information of *S. clavuligerus* ATCC 27064 genome in the reconstruction of the first genome-scale metabolic model of *S. clavuligerus* (iMM864). This model encompasses 1492 reactions, 971 metabolites, and 864 genes; it was used to simulate growth on different nitrogen and carbon sources and evaluate essentiality of the 1.8 Mb megaplasmid identified in *S. clavuligerus* ATCC 27064. Later, the same authors demonstrated that the FBA predicts the metabolic fluxes through the biosynthetic precursors of CA; in line with the gene expression data of the *S. clavuligerus* overproducer strains (*claR/ccaR* overexpression and *∆gap1*) [118]. 

In 2015, a stoichiometric model of *S. clavuligerus* (containing 100 reactions and 91 metabolites) expanded from the first model was used to explore the effect of nutrients (carbon, nitrogen, and phosphate) and oxygen limitations on CA production [44,167]. The authors found that maximization of ATP yield was the best objective function as it was the most suitable function for predicting CA production. Likewise, they found that during the phosphate limitation, CA production was favored when glutamate flux and the precursors of the urea cycle increased. 

In 2018, two model updates of iMM864 were published. First, the iLT1021 GEM containing 1494 reactions, 1360 metabolites, and 1021 genes, using ATP yield as the best objective function to represent *S. clavuligerus* cultivated in chemostat mode, while growth rate was used as the objective function for the batch cultures [165]. The authors identified the NAD+ synthase, *glyK*, and *idh* genes as the best metabolic targets for improving the CA production. In 2018, Ramirez-Malule and colleagues [43] also improved the iMM864 GEM by including gene–reaction associations and gap-filling curation. This model was used to characterize the TCA strategies used by *S. clavuligerus* at four different growth conditions associated with CA production [43]. The authors found that CA biosynthesis correlates positively with accumulation of oxaloacetate, succinate, and acetate, while it correlates negatively with accumulation of malate at different feeding conditions in continuous cultures [43]. Additionally, FBA-fluxes inferred from experimental data predicted that the phosphoenolpyruvate carboxylase activation triggers CA production. In contrast, the activation of the glyoxylate shunt (by isocitrate lyase) matched with carbon limitation conditions and a negative effect on CA titers [43]. 

The last model was updated and curated in 2019, containing 1534 reactions, 1199 metabolites and 871 genes [166]. Although this model was not validated with experimental data, it explored 18 objective functions and metabolic phenotypes of *S. clavuligerus* during production of CA and Cephamycin C.

Finally, in 2020, a new and independent reconstruction was published [19]. The topologically and thermodynamically curated iDG1237 GEM encompasses 2177 reactions, 1518 metabolites, and 1237 genes. This model was validated with experimental data and used to get insights about the metabolic reprograming strategies of *S. clavuligerus* under high and low shear stress conditions (intracellular nutrient availability). The authors showed that during phosphate-limited and low shear stress conditions the CA production halved due to the existence of overflow metabolism coincident with accumulation of TCA intermediates. These evidences suggest that GEMs constitute valuable platforms for exploring the metabolism of *S. clavuligerus* under different experimental and nutritional conditions. We invite the *S. clavuligerus* community to use these models along with multi-omics datasets for getting further insights about the regulatory links between genes, proteins and metabolism in order to improve CA production at the industrial level.

## 7. Downstream Processing of CA 

Due to its low molecular weight and its instability in aqueous solution at room temperature diverse processes for CA separation and purification have been employed, e.g., filtration or centrifugation, liquid–liquid extraction, and ion exchange chromatography. In addition to the characteristic low productivity of the bioprocess, the product yield is also compromised by the degradation of CA in aqueous phases and the efficiency of the separation steps required for the obtention of potassium clavulanate (the stable commercial form). Several authors have confirmed the degradation of CA in fermentation broths [67,69,168,169,170], synthetic buffer [68,171,172], and pure water [173,174,175]. The CA secreted by *S. clavuligerus* exhibits a high initial reaction rate (~5 h) in aqueous solution (e.g., fermentation broths) followed by a slower degradation rate in the next hours [168,169]. Degradation of CA is considered consequence of susceptibility to nucleophilic attacks at specific points. This acts as a hydrolytic mechanism in two steps: an equilibrium reaction leading to an active intermediate and an irreversible reaction of this intermediate with another CA molecule, thus leading to the degradation product [67,176,177]. As consequence, CA increases its own degradation rate when increasing concentration in the fermentation broth [68,176]. Usually, CA purification starts (after cultivation process) with the removal of the cells from cultivation broth either by centrifugation or filtration. Then, the antibiotic is separated through adsorption, liquid extraction, or both, followed by the clavulanate salt precipitation. Nevertheless, the observed instability of CA makes the efficient recovery necessary to improve the profitability of the CA production process at reduced operation costs.

One of the most explored alternatives for CA recovery from fermentation broths is the adsorption with selective materials such as ion exchange resins, activated carbon, hydrotalcites, zeolites, and double-layer hydroxides. This technique allows the biomolecules to be selectively adsorbed in solid materials while minimizing the degradation of the product. Forte et al. [178,179] studied the CA recovery from aqueous solutions by using natural and synthetic zeolites, namely, faujasite-type NaX zeolite (13X) and clinoptilolite–mordenite zeolite (NZ) and their modified cationic forms (K^+^, Na^+^, Ca^2+^, Ba^2+^, Mg^2+^, Sr^2+^), with aperture diameters ranging 0.062 and 0.150 mm. The 13X­-Na zeolite showed the best CA retention (17.4%) in the equilibrium at a zeolite/liquid ratio of 0.5 mg.g^−1^ [178]. Higher retention levels have been obtained in separations with quaternary ammonium anionic exchange resins, such as Amberlite IRA 400 and Q Sepharose XL. In this regard, Barboza et al. [180,181] studied the temperature effect on CA adsorption and desorption in flask-scale experiments, showing that CA removal from aqueous solution is very favorable (retention values between 60 and 70%) at temperatures ranging from 5 to 10 °C given the exothermicity of the CA-resin bonding. The desorption process is facilitated by higher temperatures (up to 30 °C) than the adsorption, the equilibrium time is almost doubled and the desorption ratio is about 60% [180,181]. Almeida et al. [182] proposed a simultaneous adsorption/desorption system of CA using sequential stirred tanks, allowing the recycling of the fermentation supernatants and hence, increasing the CA adsorption percentages. Absorption operations in packed columns with IRA 400 operating at a flow rate of 0.3 L∙h^−1^ and 10 °C were used, followed by desorption with 2% NaCl at 30 °C. Even though the absorption in fixed beds have provided good results in terms of separation and concentration, CA degradation may occur within the sites of the ion-exchange resin, reducing the overall yield of the process [183]. The rapid CA degradation in aqueous media has implications on the selection of the separation technology, the shorter the time of CA in aqueous solution, the lower its degradation. Furthermore, the complete CA desorption from the ion exchange matrix doubles the adsorption time, increasing the product losses. The layered double hydroxides (LDHs) have been also explored for CA adsorption from aqueous solutions. LDHs are mixed hydroxides with layers of divalent, trivalent cations, and hydroxyl anions capable of anion exchange [184]. The interlayer domain is constituted by water molecules and anions [184]. When using calcined hydrotalcite LDHs containing 70% MgO in a solid–liquid ratio of 15.0 g∙L^−1^, complete adsorption of CA was observed [184]. CA adsorption into Zn_2_Cr-NO_3_, Zn_2_Al-NO_3_, and Mg_2_Al-NO_3_ LDHs was satisfactory, but the best results were observed for the Zn_2_Cr-NO_3_ LDH microencapsulated with calcium alginate, showing a purification factor of about 2.3 and CA recovery between 93 and 99% [185,186].

Aqueous two-phase systems (ATPS) have been also studied for CA separation from fermentation broths. ATPS are formed when two hydrophilic components are mixed in water above a threshold concentration [187]. Two polymers or a polymer and a salt are commonly used. Those systems are convenient for the purification of bioactive compounds due to the high water content (70–90%) in both phases [187]. The use of ATPS for CA recovery from cultivation broths was first reported by Videira and Barros [188]. The authors used the ATPS of polyethylene glycol (PEG) and potassium phosphate for CA extraction. Under the conditions tested, CA showed high affinity to the PEG-rich (top) phase, yielding recoveries ranging from 90 to 99% [188]. The subsequent optimization studies confirmed the high recovery ratio of this technique, using PEG with molecular mass of 400 daltons at pH 6.4 and top/bottom phase volume ratios of 42 and 1.3, respectively [189]. Similar results have been observed when using other PEG based ATPS like those formed by PEG/citrate, PEG/polyacrylate and PEG/cholinium chloride [187,190,191]. Furthermore, Silva et al. [192] used ATPS followed by ion-exchange adsorption for CA separation from complex fermentation broths with high amino acids content. The ATPS-Ion exchange system produced CA recoveries of 100% in both stages, which is higher than the separation reached when using single ion-exchange adsorption. However, the CA degradation rate in ATPS systems remains to be assessed given the susceptibility of CA to hydrolysis via nucleophilic attacks to the carbonyl group [176].

Viana-Marques and co-workers [193] investigated the application of ATPS in the CA extractive fermentation with a variant of *S. variabilis* in the shake flask and bioreactor scales, taking advantage of the high extraction levels attained with this separation strategy. ATPS 25% PEG 8000 g∙mol^−1^ and phosphate salts led to CA productivity of 5.3 mg.(L∙h)^−1^ [193]. The CA concentration in the PEG-rich phase was 691 mg∙L^−1^, which was 30% higher than in the cultivation broth [193]. In the bioreactor scale (950 rpm, 3.5 vvm), this strategy allowed a maximum CA production of 434 mg∙L^−1^ [194]. The authors emphasized that this technology constitutes a low-cost platform to simultaneously produce and recover CA with high yields. Similarly, Lopes-Costa and Colli-Badino employed extractive fermentation using IRA 400 ion-exchange resin, obtaining a CA recovery of 78%; thus, increasing the cumulative CA concentration in 248% compared to the control without product removal [195]. The increase of CA productivity when it is adsorbed from the fermentation broth supports the hypothesis that high CA concentration may inhibit the antibiotic biosynthesis. 

A different approach considers the use of aqueous two-phase micellar systems (ATPMS). Those are micellar solutions that separate spontaneously in two phases: a micelle-rich phase and a micelle‑poor phase, depending on operating conditions (pH, ionic strength, and temperature) [196]. An ATPMS employing the surfactant n-decyltetraethylene oxide showed preferential partition of proteins towards the micelle-rich phase, but if a denaturation step is applied prior to extraction, the 52% of CA is recovered in this phase [196]. ATPMS formed by the non-ionic surfactant Triton X-114 (0.022%) and an anionic one (1%) allowed a CA recovery of 86.3% at 28 °C [197]. Similarly, ATPMS of dextran sulfate with non-ionic surfactants Triton X-114 or Triton X-100 have been also used for CA purification, yielding CA recoveries of 80 and 50%, respectively [198]. 

Aqueous CA from fermentation broths or concentrated solutions can also be extracted by simple liquid extraction with organic solvents. The evaluation of organic solvents butyl acetate, ethyl acetate, n-butanol, 2-butanol, and methyl isobutyl ketone showed that CA partition coefficient is related to solvent properties like dielectric constant and species solubility [199]. Although CA distribution coefficients for 2-butanol and n-butanol were found to be higher than for the esters and the methyl‑isobutyl ketone; the high solubility of light alcohols in water makes them impractical for further CA precipitation. For extraction purposes is desirable a water‑immiscible organic phase, which also reduces the degradation susceptibility of the molecule. Brites and colleagues [199] concluded that ethyl acetate is the best option considering the subsequent precipitation steps required for the formation of clavulanate salts, yielding a CA extraction of 35.6% with partition coefficient of 0.73 in acidic conditions. Furthermore, organic solvent mixtures containing methyl‑ethyl ketone/ethyl acetate and methyl‑isobutyl ketone/ethyl acetate allow higher extraction levels of CA from fermentation broths. The equivolumetric mixtures of methyl‑ethyl ketone/ethyl acetate and ethyl‑isobutyl ketone/ethyl acetate led to extraction yields of 50 and 44.7%, respectively, which is approximately 30% higher compared to the obtained for single ethyl acetate extraction [200]. 

As CA is marketed in salt form, the direct precipitation from aqueous solutions, either fermentation broths or concentrated solutions, has been studied as a purification alternative. CA extracted via organic solvents can be precipitated in two steps: (i) reaction of CA with an aliphatic amine to form a stable CA-amine salt and (ii) precipitation with potassium 2-ethyl hexanoate [201]. However, CA can be precipitated directly from the organic solvent by addition of potassium 2-ethyl hexanoate without requiring the formation of a stable intermediate [202]. However, slightly higher yield was observed when the amine intermediate is formed (72.4%) compared to the direct precipitation method (69.5%) [201,202].

## 8. Concluding Remarks

Genetic modification of genes involved, or related to, the clavam pathway leads to improvement of CA production in *S. clavuligerus.* This pathway constitutes a key point for engineering CA overproducer strains aimed to the large-scale application. Despite the high number of successful studies involving synthetic biology approaches, alternative bioprocessing strategies, identification of genes related to pathway regulation, metabolic modeling, and downstream processing, novel strategies need to be implemented to pave the way to higher CA productivity in the antibiotics industry. Thus, a multidisciplinary research approach combining microbiology, bioprocess engineering, biochemistry, molecular biology, analytical chemistry, and computational biology would help to unravel the intrinsic metabolic complexity of CA biosynthesis. The connection of CA with the reaction-steps in the clavam pathway and its relationship with the biosynthesis and release of other antibiotics like the cephamycins and penicillins should be further studied considering the interaction between the biological layers.

## Figures and Tables

**Table 1:** Studies reporting the highest CA production with engineered *S. clavuligerus* strains.

Strain	Operation Mode	Strain Intervention	Supplement	Titers (g∙L^−1^)	Ref.
*S. clavuligerus* IDG3	Fed-batch	Overexpression: *cas2*, *ccaR*, *claR*	Glycerol trioleate	6.690	[95]
*S. clavuligerus* M3-19	Batch	Reporter gene neo fused downstream of *claR*	-	4.330	[96]
*S. clavuligerus* NEO	Batch	Reporter gene neo fused downstream of *claR*	-	3.260	[96]
*Streptomyces sp. MU-NRC77*	Fed-batch/Batch	UV mutagenesis	H_2_O_2_/Activated animal charcoal	0.649/0.683	[119]
*S. clavuligerus* 70	Batch	UV mutagenesis	-	0.500	[120]
*S. clavuligerus* OR	Batch	Random mutagenesis and *cas1*, *ccaR* and *claR* overexpression	-	5.520 (OR/pCAS1) 6.010 (OR/pCCAR-CLAR)	[97]
